# Peer assessment of outpatient consultation letters – feasibility and satisfaction

**DOI:** 10.1186/1472-6920-7-13

**Published:** 2007-05-22

**Authors:** Erin Keely, Kathryn Myers, Suzan Dojeiji, Craig Campbell

**Affiliations:** 1 Department of Medicine, University of Ottawa, Canada; 2 Department of Medicine Queen's University of Ottawa, Canada

## Abstract

**Background:**

Written correspondence is one of the most important forms of communication between health care providers, yet there is little feedback provided to specialists. The objective of this study was to determine the feasibility and satisfaction of a peer assessment program on consultation letters and to determine inter-rater reliability between family physicians and specialists.

**Methods:**

A rating scale of nine 5-point Likert scale items including specific content, style items, education value of the letter and an overall rating was developed from a previous validated tool.

Nine Internal Medicine specialists/subspecialists from two tertiary care centres submitted 10 letters with patient and physician identifiers removed. Two Internal Medicine specialists, and 2 family physicians from the other centre rated each letter (to protect writer anonymity). A satisfaction survey was sent to each writer and rater after collation of the results. A follow-up survey was sent 6–8 months later.

**Results:**

There was a high degree of satisfaction with the process and feedback. The rating scale information was felt to be useful and appropriate for evaluating the quality of consultation letters by 6/7 writers. 5/7 seven writers felt that the feedback they received resulted in immediate changes to their letters. Six months later, 6/9 writers indicated they had maintained changes in their letters.

Raters rank ordered letters similarly (Cronbach's alpha 0.57–0.84) but mean scores were highly variant. At site 1 there were significant differences in scoring brevity (p < 0.01) between family physician and specialist raters; whereas, at site 2 there were differences in scoring of history (p < 0.01), physical examination (p < 0.01) and educational value (p < 0.01) of the letter.

**Conclusion:**

Most participants found peer assessment of letters feasible and beneficial and longstanding changes occurred in some individuals. Family physicians and specialists appear to have different expectations on some items. Further studies on reliability and validity, with a larger sample, are required before high stakes professional assessments include consultation letters.

## Background

Written correspondence is an important form of communication between primary care physicians and consultants. Poor communication may result in delayed diagnosis, inadequate follow-up, erosion of patient confidence and increased costs through duplication of services [1]. Although competency in written communication is essential, most Canadian physicians have not received any training or feedback about their letters [2,3]. Peer evaluation of consultation letters could add valuable feedback to practicing physicians.

The consultation letter reflects the diagnostic skills, communication skills, professionalism, and charting management of a physician. It requires synthesis of clinical data, but also reflects distribution of responsibility between providers, professional courtesy, legal requirements, and the writer's ability to educate regarding a specific case [3]. Consultation letters are generally accessible to review and are written to be read by others, making them ideal for peer assessment. Audits may be used to promote changes in practice based on an assessment of actual performance in relation to some standard of care. An audit requires the use of an audit tool, the collection and analysis of actual performance in relation to best evidence or practice, and finally the identification of an outcome(s) for practice [4].

The objectives of this pilot study were to:

1. develop, implement and evaluate the feasibility and acceptability of a peer assessment program for practicing physicians' written consultation letters

2. determine inter-rater reliability of a novel practice audit tool for consultation letters.

## Methods

### 1. Development of the audit tool

**Figure 1:**
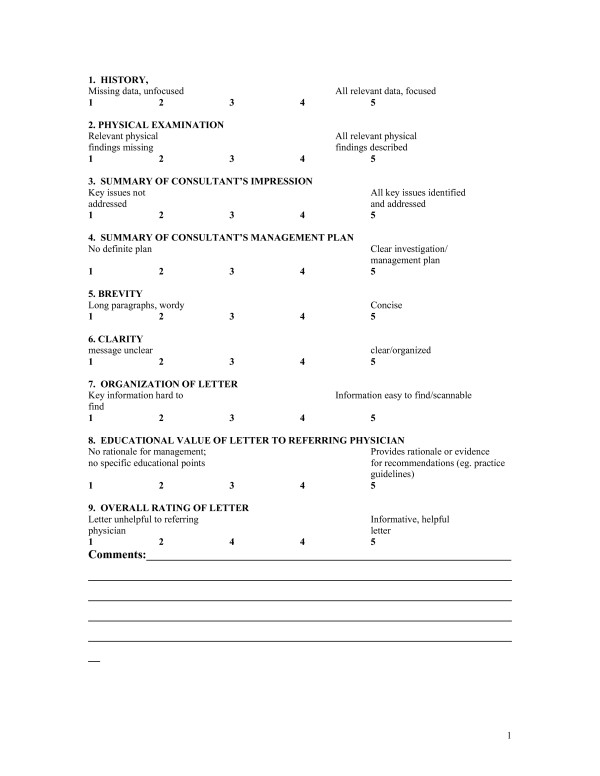
Consultation Letter Rating Scale.

Previous work on the development of an evaluation tool for residents' letters provided the background for the design of an audit tool for practicing physicians [5,6]. To ensure face validity, content and style items were generated from surveys of physicians, literature review and advice from written communication experts. The original tool, a 34 item scale, was revised based on a pilot study with trainee letters [5]. The dichotomous variables were removed because of low inter-rater reliability and likert scale items were reduced to avoid repetition. The revised scale consists of 9 five point likert scales with anchors to help guide raters (figure 1).

### 2. Collection and rating of letters

Internal Medicine specialists, actively engaged in clinical practice, were recruited through grand rounds, email and personal contact from two tertiary care institutions in two cities. Each was asked to submit 10 consecutive consultation letters. Two family physicians and two internal medicine specialists, identified as individuals interested in improving written communication but not involved in the development of the rating scale, were enlisted from each site to be raters (total 8 raters). To provide anonymity of the writer, each rater only evaluated letters from the other site.

All patient and physician identifiers were removed by the writer and the letters sent to a central location. The letters were checked for blinding, coded and then sent to the raters with the rating scale. Each rater evaluated all letters from the writers at the other site. All writers were also asked to self-assess their letters using the same scale.

### 3. Feedback

All rating scales were returned to a central administrator. The scores and comments from the 4 raters and the self-assessment scores were collated and provided to the writer. All writers and raters were asked to provide written feedback on the usefulness and logistics of the audit process. A 16-item 5-point likert scale item survey, using a 5 point likert scale (strongly agree to strongly disagree) was used for raters and a 19 item survey for writers. Items included the blinding of the letters, usability of the scale, satisfaction with feedback provided and feasibility of a nationally organized program. A follow-up email survey was sent to all writers 6–8 months later asking 2 questions: were specific changes made to the content and style of letters as a result of the feedback they received (asked to specify) and were their letters taking more, less or the same amount of time to produce.

The research ethics boards at both institutions approved this study. Participants signed an informed consent.

### 4. Statistical analysis

Descriptive statistics were generated for each item for each writer. An overall score was calculated for each letter by summing the nine-likert items. Responses to the surveys were tabulated.

Inter-rater reliability was assessed two ways. Using each letter as the unit of analysis, Cronbach's alpha was calculated to look at average correlation between raters on total score. To assess variance between raters' scores, intraclass correlation coefficients (ICC) were used. If raters rank order letters the same way, but the mean ratings are markedly different, there will be a discrepancy between the two methods. If the Cronbach's alpha is higher than the ICC then it means raters rank order letters fairly similarly but have different mean ratings.

To compare family physician vs. specialist ratings, differences between mean scores for each item and overall letter score were compared using ANOVA. The two sites were treated independently as they were a unique group of raters and unique group of writers.

## Results

Nine specialists, 4 from one site and 5 from the other, submitted 10 letters each. The mean scores for each item are shown in table 1.

**Table 1:** Differences in mean scores of each item between family physicians (n = 2 per site) and specialists (n = 2 per site)

**I. Site 1 (n = 4 writers with 10 letters each)**			
Item	GP	Specialist	p

History	4.04 ± 1.0	4.16 ± 0.95	NS
Physical Examination	4.34 ± 0.90	4.23 ± 0.98	NS
Impression	4.12 ± 0.90	4.14 ± 1.0	NS
Plan	4.21 ± 0.85	4.21 ± 0.99	NS
Brevity	3.87 ± 0.94	4.25 ± 0.87	0.003
Clarity	4.00 ± 1.1	4.14 ± 1.0	NS
Format	3.98 ± 1.1	4.01 ± 1.0	NS
Educational Value	4.02 ± 0.95	3.90 ± 0.90	NS
Global rating	4.04 ± 0.96	3.92 ± 0.97	NS
Overall score	36.45 ± 7.36	36.97 ± 7.85	NS

**II Site 2 (n = 5 writers with 10 letters each)**			

Item	GP	Specialist	p

History	3.88 ± 1.1	4.40 ± 0.72	0.0001
Physical Examination	3.90 ± 0.97	4.45 ± 0.71	<0.0001
Impression	4.00 ± 1.0	4.08 ± 0.96	NS
Plan	4.14 ± 0.90	4.18 ± 0.87	NS
Brevity	3.64 ± 1.2	3.83 ± 0.90	NS
Clarity	3.931 ± .0	4.03 ± 0.91	NS
Format	3.63 ± 1.1	3.68 ± 0.67	NS
Educational Value	4.00 ± 1.0	3.38 ± 1.1	<0.0001
Global rating	3.88 ± 0.93	3.74 ± 0.91	NS
Overall score	34.97 ± 7.70	35.79 ± 5.19	NS

The satisfaction surveys were returned from 7/9 writers and 8/10 raters. All writers agreed or strongly agreed that the process of collecting, blinding and submitting letters was manageable and that the rating scale items were valid. The rating scale information was felt to be useful and appropriate for evaluating the quality of consultation letters by 6/7 writers. Five of seven writers felt that the feedback they received resulted in immediate changes to their letters. The eight raters that replied all agreed that they received the letters and rating scales in an organized manner with clear instructions and blinding. 7/8 agreed that the rating scale length was adequate, easy to use and not too time demanding. Six of eight raters agreed the rating scale was an appropriate method of evaluating letters.

All writers replied to the 6–8 month follow-up email survey. Three of the nine did not make any significant changes to their letters. Six of the nine made long-lasting changes which included using more headings (n = 2), clear identification of reason for referral and recommendations (n = 3), more specific recommendations in drug therapy/treatment targets (n = 3), avoiding irrelevant details (n = 3), and use of point form or bullets (n = 1). For three respondents, their letters took more time; whereas, one noted a decrease in the time required.

The assessment of inter-rater reliability was divided into the two sites as they were distinct groups of raters, writers and letters. Cronbach's alpha between raters for overall letter score was 0.84 for site 1 and 0.57 for site 2. Intra-class correlation coefficients for individual letter items and overall score, ranged from 0.19–0.39 for site 1 and 0.08–0.25 for site 2. Thus inter-rater reliability as assessed by Cronbach's alpha was significantly higher than those determined by intra-class correlation coefficients.

When family physicians were compared to specialist ratings there was a significant difference in scoring for brevity in site 1 and for history, physical examination and educational value for site 2. (see table 1)

## Discussion

Our study shows that a peer assessment program using a nine item rating scale is a feasible form of practice audit for consultation letters. Participating physicians were satisfied with the audit, appreciated the feedback and most participants demonstrated self-perceived long-lasting changes in their consultation letters.

The opportunity for reflection and self-improvement is the goal of an audit. Many of our writers made on-going changes to their letters as a result of this study. Other studies have also demonstrated improvement secondary to feedback. Fox et al evaluated 15 letters from 5 pediatric consultants, each rated by 1 GP, and 1 pediatric registrar[7]. Three months later, all but one participant showed improvement in overall score. Tattersall, compared letters from 31 oncologists before and after attending a training program which included feedback on their own letters, specific recommendations for content and style of letters, and a prompt card to help with further dictation [8]. There were significant improvements in use of problem lists, headings, and inclusion of specific content items. Thus, all evidence suggests that feedback does result in change in consultation letters.

Our evaluation of inter-rater reliability demonstrated reasonable consistency in rank ordering of letters, however, there was a high degree of variance between raters in actual score. Potential factors for this high variance include the limited training of the raters on scale items, and different opinions of what constitutes an ideal letter. Although there is consensus in the literature on recommended style and content of letters, there is no consensus on minimum standard that could be applied to high stake reviews. The discrepancies between raters in our study, requires that further work on criteria and standards of performance be developed prior to using letters in high stakes re-certification programs.

There were significant differences between family physician and specialist ratings on some items. Letters serve both as correspondence with the referring practitioner, but also are the main record keeping tool for the specialist [9]. These different roles for consultation letters may impact on the expectations between these two professional groups. The importance of brevity and educational value is likely better judged by the family physician whereas history and physical examination sections would be important to both groups. Westerman took three random referrals from internal medicine, dermatology, neurology and gastroenterology clinics per month and had 4 family physicians and 4 specialists rate them [10]. They had similar poor inter-observer agreement between specialists and family physicians. As in our study, family physicians were more positive about the educational value of the letters than specialists.

Other models for peer-assessment of consultation letters may be more practical outside of a study setting. Rating scales could be attached to the letter as it is sent to the referring physician. This would negate the need for blinding (as the physician is already the recipient of the letter) and may increase the likelihood of change as the feedback is from the person directly responsible for the implementation of the recommendations in the letter. Although specialist peer feedback would not be available, the referring physician, as the primary consumer of consultation letters, is likely the more valuable rater of performance assessment.

Our study was a pilot project with a small sample of writers limited to Internal Medicine. The two site model, for anonymity of writers, resulted in increased variability by having two groups of raters, which performed quite differently. The low inter-rater reliability could likely be improved by sampling a larger number of letters and increasing the number of raters. However, the overall satisfaction with the formative feedback and ease of use of the instrument are perhaps as important in this setting as the psychometric properties.

## Conclusion

Consultation letter writing is an essential skill for practicing specialists. The lack of feedback and education during training, make it a good target for continuing professional development. Peer feedback and self-reflection resulted in long-lasting changes in some individuals. Further studies on reliability and validity, with a larger sample, are required.

## Competing interests

The author(s) declare that they have no competing interests.

## Authors' contributions

EK originated the study design. She was responsible for collection of data, interpretation of data and drafted the manuscript. KM and SD participated in design of study, acquisition of data and interpretation of data. KM and SD revised the draft of the manuscript. CC participated in study design and interpretation of data. CC revised the draft of the manuscript. All authors have given final approval of the version to be published.

## Pre-publication history

The pre-publication history for this paper can be accessed here:


